# SGFSC: speeding the gene functional similarity calculation based on hash tables

**DOI:** 10.1186/s12859-016-1294-0

**Published:** 2016-11-04

**Authors:** Zhen Tian, Chunyu Wang, Maozu Guo, Xiaoyan Liu, Zhixia Teng

**Affiliations:** 1 School of Computer Science and Technology, Harbin Institute of Technology, Harbin, 150001 People’s Republic of China; 2 Department of Information Management and Information System, Northeast Forestry University, Harbin, 150001 People’s Republic of China

**Keywords:** Gene ontology, Hash table, Gene functional similarity

## Abstract

**Background:**

In recent years, many measures of gene functional similarity have been proposed and widely used in all kinds of essential research. These methods are mainly divided into two categories: pairwise approaches and group-wise approaches. However, a common problem with these methods is their time consumption, especially when measuring the gene functional similarities of a large number of gene pairs. The problem of computational efficiency for pairwise approaches is even more prominent because they are dependent on the combination of semantic similarity. Therefore, the efficient measurement of gene functional similarity remains a challenging problem.

**Results:**

To speed current gene functional similarity calculation methods, a novel two-step computing strategy is proposed: (1) establish a hash table for each method to store essential information obtained from the Gene Ontology (GO) graph and (2) measure gene functional similarity based on the corresponding hash table. There is no need to traverse the GO graph repeatedly for each method with the help of the hash table. The analysis of time complexity shows that the computational efficiency of these methods is significantly improved. We also implement a novel Speeding Gene Functional Similarity Calculation tool, namely SGFSC, which is bundled with seven typical measures using our proposed strategy. Further experiments show the great advantage of SGFSC in measuring gene functional similarity on the whole genomic scale.

**Conclusions:**

The proposed strategy is successful in speeding current gene functional similarity calculation methods. SGFSC is an efficient tool that is freely available at http://nclab.hit.edu.cn/SGFSC. The source code of SGFSC can be downloaded from http://pan.baidu.com/s/1dFFmvpZ.

## Background

In the functional genomic era, measuring gene functional similarity is a fundamental task because it is the foundation of much essential research such as gene clustering [1–4], protein-protein interaction prediction [5–8], gene function prediction [9–12] and disease gene prioritisation [13–15]. Comparing functional similarity between genes provides more information for understanding the biological roles and functions of genes, although sometimes it may be less objective compared with sequence and structure similarity [16].

Gene Ontology (GO) is a standardised and controlled vocabulary of terms that comprises three orthogonal ontologies: cellular component (CC), molecular function (MF) and biological process (BP). These three ontologies are structured as three directed acyclic graphs (DAGs), which are also called GO graphs sometimes. Semantic similarity applied to the GO annotations of gene products provides a measure of their functional similarity. Therefore, functional similarity between genes can be inferred from the semantic relationships of GO terms. In this article, ‘functional similarity’ refers to the similarity between genes or gene products, and ‘semantic similarity’ refers to the similarity between two GO terms.

In recent years, many gene functional similarity calculation measures [15, 17–30] have been proposed and widely used in biology research. They are mainly divided into two categories: pairwise approaches and group-wise approaches, both of which must rely on GO graphs [31]. Pairwise methods measure the gene functional similarity via two steps. The first step computes the semantic similarities of GO term pairs using term comparison techniques. The second step measures gene functional similarity between genes using the results of semantic similarity scores from the first step. Maximum rule, average rule and best-match average rule (BMA) are three kinds of strategies widely used in the second step. The characteristics of the three rules have been discussed in detail [16, 17]. In contrast, group-wise methods measure gene functional similarity by comparing the terms that annotate the genes in groups. Overall, there are three types of approaches for measuring the functional similarities of genes: set, graph and vector [31].

However, the common issue of current functional similarity calculation methods is time consumption, especially when they measure similarity on a whole genomic scale. There are two main reasons for the low computational efficiency, which becomes very prominent. One is that more and more GO terms are added into the GO graphs because of the daily evolution of the GO database. The other is that the number of annotated genes in the Gene Ontology Annotation (GOA) database has greatly increased. Although some online tools [23, 25, 32–36] have achieved great success on a variety of applications such as constructing gene semantic similarity networks [37, 38] and disease gene prioritisation [39–41], few of them pay attention to the problem of computational efficiency. Thus, improving the computational efficiency of functional similarity methods has become a challenging problem.

In the remainder of this section, we review seven typical methods that use the proposed strategy and some other methods. These methods include those of Resnik [20], Lin [19], Jiang and Conrath (hereafter referred to as Jiang) [18], Pekar and Staab (hereafter referred to as Pekar) [30] and Wang et al. (hereafter referred to as Wang) [21], which are pairwise approaches, and simUI [29] and simGIC [23], which are group-wise approaches. It should be noted that we emphasise the analysis of semantic similarity between terms for pairwise approaches and highlight functional similarity between genes for group-wise approaches.

The methods of Resnik, Lin and Jiang belong to node-based approaches, which rely on comparing the properties of terms in the GO graph. One concept commonly used in these approaches is information content (IC), which gives a measure of how specific and informative a term is. The IC of a term *t* can be quantified as the negative log likelihood:

1 $$ IC(t)=- \log \left(\Big(p(t)\right) $$

where p (*t*) is the probability of occurrence of *t* in a specific corpus (such as the UniProt knowledgebase) being normally estimated by its frequency of annotation [31]. According to Eq. (1), terms that are more genetic will have a larger p (*t*) and hence a smaller IC value. When applying this traditional measure, one important problem is that the specificity of a term is fully dependent on the number of genes taken in a given annotation corpus [24]. The detail definition of p (*t*) can be represented by Eq. (2).

2 $$ p(t)=\frac{annotation(t)+{\displaystyle {\sum}_{d\in descendent(t)}\left( annotation(d)\right)}}{{\displaystyle {\sum}_{c\in descendent(root)}\left( annotation(c)\right)}} $$

Here, annotation (*t*) is the number of genes annotated by term *t*, and descendent (*t*) is a term set that contains descendants of *t* in the GO graph.

Resnik [21] elaborated that edges do not represent the same uniform distance in the GO graph because the terms at the same level do not have the same specificity. Some terms in the GO graph have more children even though they belong to the same level. Therefore, an IC value of the term *t* in the GO graph can be used as a metric to measure the relationship between terms. Resnik defined the similarity between two terms *t*_1_ and *t*_2_ as the IC value of the lowest common ancestor term (LCA), which was given as follows:

3 $$ si{m}_{Resnik}\left({t}_1,{t}_2\right)=IC\left(LCA\left({t}_1,{t}_2\right)\right) $$

According to Eq. (3), the similarity between two terms only depends on the IC value of their LCA. Sometimes, LCA is also called the most informative common ancestor.

Lin [19] pointed out one serious drawback of the Resnik method, which is that two different pairs of terms that locate on different levels in the GO graph will have the same LCA, so they have the same similarity value. Apparently, this is not a reasonable result that meets the human perspective. Therefore, both Lin and Jiang invented two other measures, which are formulated as Eqs. (4) and (5), respectively:

4 $$ si{m}_{Lin}\left({t}_1,{t}_2\right)=\frac{2\ast IC\left(LCA\left({t}_1,{t}_2\right)\right)}{IC\left({t}_1\right)+IC\left({t}_2\right)} $$

5 $$ si{m}_{JC}\left({t}_1,{t}_2\right)=1-\left(IC\left({t}_1\right)+IC\left({t}_2\right)-2\times IC\left({t}_{LCA}\right)\right) $$

As is pointed out by Wang [21], the methods of both Lin and Jiang have the problem of “shallow annotation”, i.e. if two genes are well annotated near the root of the ontology, their semantic similarity will always be measured very highly (close to 1), and their semantic distance will always be computed close to nil, thus providing a misleading result.

In contrast, edge-based approaches are also popular in measuring semantic similarity between GO terms. Pekar [30] proposed a measure based on the length of the longest path between the lowest common ancestor of two terms and the root, and on the length of the longest path between each term and their common ancestor. This is given by the following expression:

6 $$ si{m}_{PS}\left({t}_1,{t}_2\right)=\frac{\delta \left({t}_a, root\right)}{\delta \left({t}_a, root\right)+\delta \left({t}_1, root\right)+\delta \left({t}_2, root\right)} $$

where *δ*(*t*_1_, *t*_2_) denotes the longest distance between term *t*_1_ and term *t*_2_ in the GO graph, and *t*_a_ is the LCA of *t*_1_ and *t*_2_. Three distances are used in Eq. (6), and thus the functional similarity computed by this method is more reasonable than that of Resnik’s results. In addition, Cheng et al. [27] proposed a maximum common ancestor depth measure and weighted each edge to reflect its depth. Wu et al. [26] introduced the distance to the nearest leaf node of a term and the distance to the LCA to take the specificity of terms into account.

Wang [21] developed a hybrid method in which the edge was assigned a fixed weight named the semantic contribution factor (*ω*_*e*_) according to the type of relationship in the GO database. A GO term *A* is represented as a DAG *DAG*_*A*_ = (*A*, *T*_*A*_, *E*_*A*_), a sub-graph of GO where *T*_A_ is the set of all ancestors for term *A*, and *E*_*A*_ is the set of corresponding links. The contribution of any term *t* to the semantics of a term *A* is defined as the S-value of the term *t* related to term *A*, which can be represented by

7 $$ \left\{\begin{array}{l}{S}_A(t)=1\kern14.25em \mathrm{if}\kern0.75em t = A\\ {}{S}_A(t)= \max \left\{{w}_e\ast SA\left({t}^{\prime}\right)\left|{t}^{\prime}\in chilrenof(t)\right.\right\}\kern1.25em \mathrm{if}\kern0.75em t\ne A\end{array}\right. $$

where (*ω*_*e*_) is the semantic contribution factor for edge e∈E_A_ linking term *t* with its child term *t*′ [23]. Then, we calculate the semantic value of the GO term *A*, *SV*(*A*), which is represented as:

8 $$ SV(A)={\displaystyle \sum_{t\in {T}_A}{S}_A(t)} $$

Given *DAG*_*A*_ = (*A*, *T*_*A*_, *E*_*A*_) and *DAG*_*B*_ = (*B*, *T*_*B*_, *E*_*B*_) for GO terms *A* and *B*, respectively, the semantic similarity between them, *S*_*GO*_(*A*, *B*), is defined as:

9 $$ {S}_{GO}\left(A,B\right)=\frac{{\displaystyle \sum_{t\in {T}_A\cap {T}_B}\left({S}_A(t)+{S}_B(t)\right)}}{SV(A)+SV(B)} $$

where *S*_*A*_(*t*) is the S-value of GO term *t* related to term *A*, and *S*_*B*_(*t*) is the S-value of GO term *t* related to term *B*. There are two main disadvantages of Wang’s method. One is that the semantic contribution factor (*ω*_*e*_) is fixed according the linking types of GO terms, and the other is that the semantic contribution only depends on the maximum products of all of the paths linking the two terms. According to Eqs. (7) and (8), computing the SV(*A*) and SV(*B*) is difficult because they have to traverse their corresponding DGAs of term *A* and *B*, respectively.

As for group-wise approaches, Pesquita et al. [31] pointed out that purely set-based approaches are not common because few measures consider only direct annotations, whereas graph-based approaches are suitable for computing the similarity with the help of graph matching. Indeed, simUI and simGIC are two typical group-wise methods that measure gene functional similarity from the graph-based perspective.

To compute gene functional similarity, these methods usually make use of Tversky’s ratio model or its variants. Genes *g*_1_ and *g*_2_ are annotated with term sets *A*_*g*1_ = {*t*_1_, *t*_2_, ⋯ *t*_*m*_} and *A*_*g*2_ = {*t*_1_, *t*_2_, ⋯ *t*_*n*_}, respectively. Therefore, simUI calculates similarity as the number of GO terms shared by two genes divided by the number of GO terms they have together. The functional similarity between *g*_1_ and *g*_2_ is

10 $$ simUI\left({g}_1,{g}_2\right)=\frac{\left|{A}_{g1}\cap {A}_{g2}\right|}{\left|{A}_{g1}\cup {A}_{g2}\right|} $$

According to [42], simGIC is an expansion of simUI that sums the IC value of annotation terms. For two genes *g*_1_ and *g*_2_, simGIC is given by

11 $$ simGIC\left({g}_1,{g}_2\right)=\frac{{\displaystyle {\sum}_{t_i\in {A}_{g1}\cap {A}_{g2}}IC\left({t}_i\right)}}{{\displaystyle {\sum}_{t_j\in {A}_{g1}\cup {A}_{g2}}IC\left({t}_j\right)}} $$

Although simUI does not consider the specificity of the term in the GO graph, simGIC takes the IC value of a term as its specificity. As pointed out by Teng et al. [16], simGIC ignores the shared IC value of two terms in the GO graph and may also result in misjudgements of gene functional similarity. Teng et al. [16] proposed a new method called SORA (semantic overlap ration of annotation) to overcome the limitations of simGIC. However, obtaining A(g_1_) and A(g_2_) from the GO graph directly for group-wise methods is also difficult and time consuming.

The rest of this paper is organised as follows. In the Methods section, we begin by analysing the problems leading to high time consumption of each method. In the following subsections, we describe how to speed the gene functional similarity calculation methods based on hash tables. Then, taking Wang’s method as an example, we show how to establish the hash table and measure the functional similarity with the table. Finally, complexity analysis is presented for each method. In the Results section, we present the experimental results, including the running times to establish hash tables and measure the semantic similarity between GO terms and the functional similarity between genes. We also compare the developed Speeding Gene Functional Similarity Calculation tool (SGFSC) with other online tools. In the Discussion section, we discuss the implications and limitations of our method, and finally, we draw our conclusions in the Conclusions section.

## Methods

In this section, we first analyze what factors affect computational efficiency and establish a hash table to speed the gene functional similarity calculation for each method. Then we take Wang’s method as an example to show how to speed the computing process using the proposed strategy. Finally, we present a complexity analysis for each method adopting our proposed strategy.

### Analysing the problems leading to time consumption for each method

Methods that measure gene functional similarity must traverse the GO graph repeatedly to obtain the information they need. However, traversing the GO graph is time consuming because the topological structure of the GO graph is complex due to multiple inheritances of GO. In addition, the problem of low computational efficiency tends to be more prominent, especially when gene functional similarity needs to be measured on the genomic scale.

To speed the gene functional similarity calculation, we should analyse the calculation process of each method and then determine the key information that affects the computational efficiency. It is need to calculate key information that lead to traversing the GO graph repeatedly for these methods. The key information for each method is listed in Table 1. IC(*t*) denotes the IC value of term *t*; LCA(*t*_1_, *t*_2_) denotes the lowest common ancestor of terms *t*_1_ and *t*_2_; Dep(*t*) denotes the deepest depth of term *t* in the GO graph; SV(*t*) denotes the semantic value of term *t*; and A(*g*) denotes the term set containing all of the terms that annotate gene *g*.

**Table 1:** Key information affecting computational efficiency for each method

Methods	Key information affecting computational efficiency
Resnik	LCA(*t*_1_,*t*_2_) and IC(LCA(*t*_1_,*t*_2_))
Lin	LCA(*t*_1_,*t*_2_) and IC(LCA(*t*_1_,*t*_2_))
Jiang	LCA(*t*_1_,*t*_2_) and IC(LCA(*t*_1_,*t*_2_))
Pekar	LCA(*t*_1_,*t*_2_) and Dep (LCA(*t*_1_,*t*_2_))
Wang	SV(*t*_1_) and SV(*t*_2_)
simUI	A(*g*_1_) and A(*g*_2_)
simGIC	A(*g*_1_) and A(*g*_2_)

For pairwise approaches, we focus on the semantic similarity between *t*_1_ and *t*_2_. For group-wise approaches, the functional similarity between gene *g*_1_ and *g*_2_ requires special attention

For example, with the Resnik method, determining the LCA of terms *t*_1_ and *t*_2_ from the GO graph requires traversing of the DAGs of *t*_1_ and *t*_2_, respectively. The method also has to calculate the IC of each term in the DAGs to obtain the IC of LCA(*t*_1_, *t*_2_). Therefore, the key information that affects computational efficiency in the Resnik method is LCA(*t*_1_, *t*_2_) and IC(LCA (*t*_1_, *t*_2_)). The key information for the other methods is also listed in Table 1.

### Speeding the gene functional similarity calculation for each method

As we know, traversing the GO graph repeatedly is the main reason for the reduced computational efficiency of each method. Therefore, if we can avoid traversing the GO graph repeatedly, the computational efficiency for each method will improve greatly. Hence, we can convert the storage form of information from the GO graph into a hash table. Then, these methods could measure the functional similarity based on the hash tables instead of traversing the GO graph, eventually reaching the goal of improving computational efficiency.

**Fig. 1:**
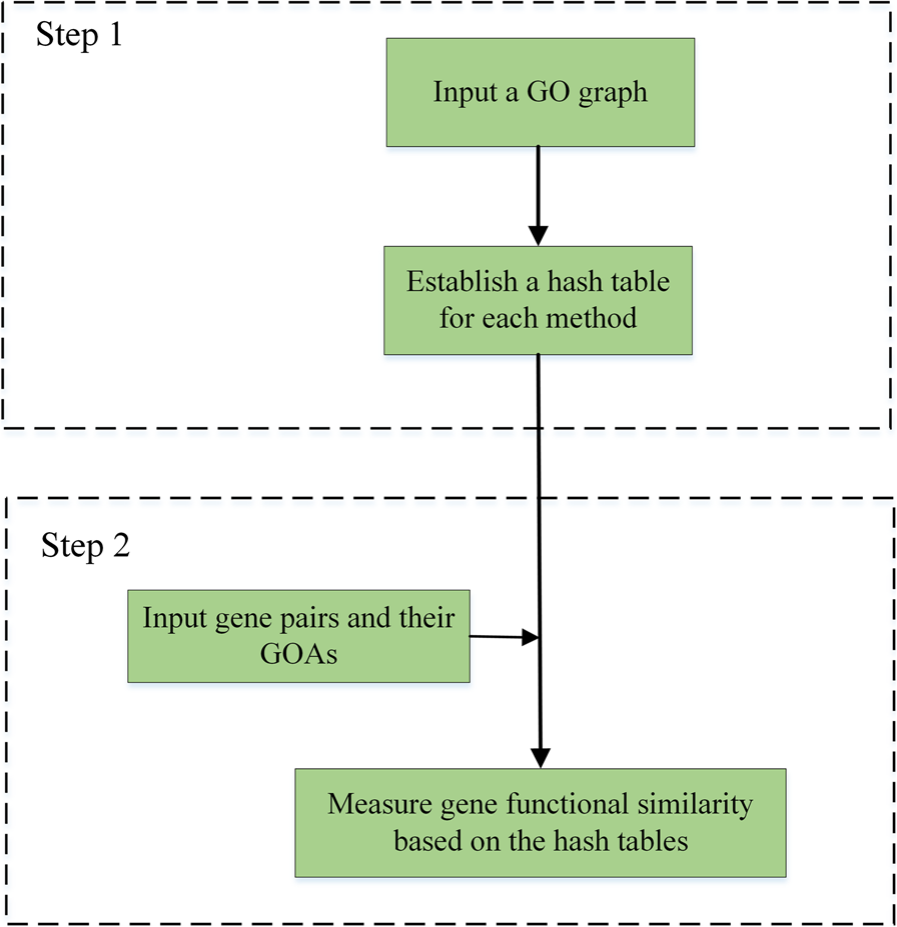
The flowchart of the proposed strategy

As a result, we propose a novel two-step computing strategy: (1) establish hash tables to store essential information that is obtained from the GO graph and (2) measure gene functional similarity based on the hash tables. The flowchart of our proposed strategy is shown in Fig. 1. For ease of description, two definitions are given below.

DEFINITION 1. **Direct information**: information that occurs in corresponding equations for each method. For example, the IC values of *LCA*(*t*_1_, *t*_2_), *IC*(*t*_1_) and *IC*(*t*_2_) are direct information for Eq. (4).

DEFINITION 2. **Essential information**: information that will be stored in the corresponding hash tables. For example, the ancestors of term *t*_1_, *t*_2_ and their corresponding IC values are essential information for Eq. (4).

It is important to note that the essential information should have the ability to substitute the GO graph. This is critical for the proposed strategy. Therefore, before using our proposed strategy, each method must first analyse the direct information and extract the essential information from the original GO graph.

The direct information and essential information of pairwise approaches for measuring semantic similarity between *t*_1_ and *t*_2_ are shown in Table 2. *T*(*t*) denotes the ancestor set of term *t* including *t* itself. *S*_*A*_(*t*) is the S-value of GO term *t* related to term *A*. The direct information and essential information of group approaches for measuring functional similarity between genes *g*_1_ and *g*_2_ are also shown in Table 2. A(g) denotes a term set containing all of the terms that annotate gene *g*. In the next subsection, we take the Wang method as an example to show how to establish the hash table for each method.

**Table 2:** Direct information and essential information for each method

Method	Direct information	Essential information	Explanation
Resnik	IC(LCA(*t*_1_,*t*_2_))	IC(*t*), *t* ∈ *T*(*t*_1_), IC(*t*), *t* ∈ *T*(*t*_2_)	The IC values of *t*, *t* ∈ *T*(*t*_1_) The IC values of *t*, *t* ∈ *T*(*t*_2_)
Lin	IC(*t*_1_), IC(*t*_2_), IC(LCA(*t*_1_,*t*_2_))		
Jiang	IC(*t*_1_), IC(*t*_2_), IC(LCA(*t*_1_,*t*_2_))		
Pekar	Dep (LCA (*t*_1_, *t*_2_)), Dep(*t*_1_), Dep(*t*_2_)	Depth (*t*), *t∈ T*(*t*_1_) Depth (*t*), *t∈ T*(*t*_2_)	The depth of *t*, *t* ∈ *T*(*t*_1_) The depth of *t*, *t* ∈ *T*(*t*_2_)
Wang	∑(*S*_*t*1_(*t*) + *S*_*t*2_(*t*)), *t* ∈ *T*_*t*1_ ∩ *T*_*t*2_ *SV*(*t*_1_), *SV*(*t*_2_)	$$ \begin{array}{l}{S}_{t_1}(t),t\in T\left({t}_1\right)\\ {}{S}_{t_2}(t),t\in T\left({t}_2\right)\end{array} $$	The S-values of *t*, *t* ∈ *T*(*t*_1_) The S-values of *t*, *t* ∈ *T*(*t*_2_)
simUI	|*A*(*g*_*i*_) ∩ *A*(*g*_*j*_)|, |*A*(*g*_*i*_) ∪ *A*(*g*_*j*_)|	*A*(*g*_*i*_), *A*(*g*_2_)	
simGIC	$$ \begin{array}{l}{\displaystyle \sum IC(t),t}\in A\left({g}_i\right)\cap A\left({g}_j\right)\\ {}{\displaystyle \sum IC(t),t}\in A\left({g}_i\right)\cup A\left({g}_j\right)\end{array} $$	$$ \begin{array}{l}IC\left({t}_1\right),{t}_1\in A\left({g}_i\right)\\ {}IC\left({t}_2\right),{t}_2\in A\left({g}_j\right)\end{array} $$	The IC values of *t*, *t* ∈ *A*(*g*_*i*_) The IC values of *t*, *t* ∈ *A*(*g*_*j*_)

Finally, why do we select the hash table as the data structure to store the essential information extracted from the GO graph? A hash table is a commonly used data structure that satisfies the requirement for quick searches. Its search efficiency is very high, and its structure is also convenient to program and implement. What’s more, with the help of the hash tables, there is no need to repeatedly traverse the GO graph to obtain direct information. Each method can obtain essential information from the hash table and calculate the direct information by making use of the essential information. As a result, the computational efficiency increases dramatically.

### Speeding the calculation of functional similarity for the Wang method

**Fig. 2:**
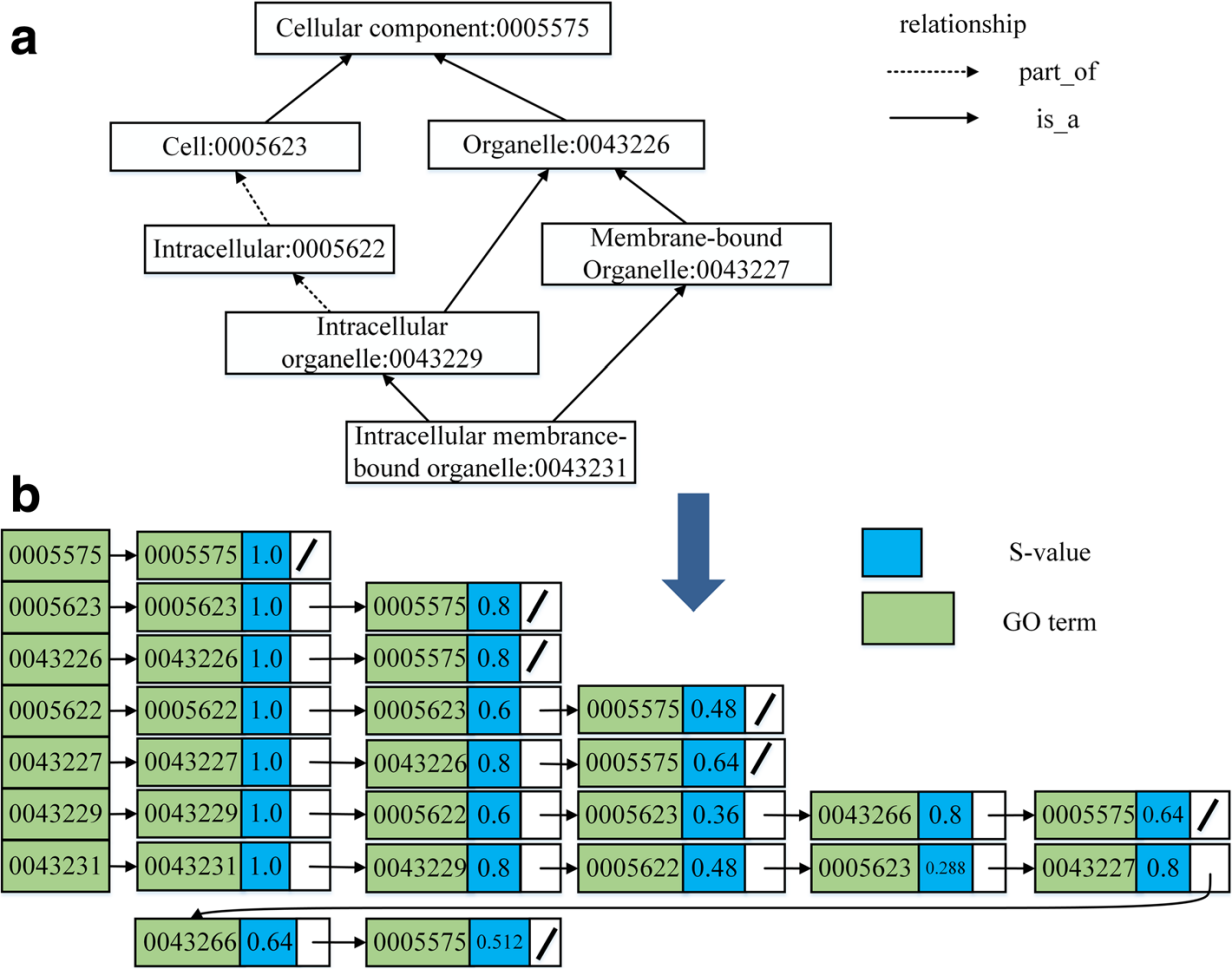
The main idea of the proposed strategy adopted for the Wang method. **a** Depicts a DAG for GO term *Intracellular Membrane*-*bound Organelle*: 0043231. **b** Depicts the hash table established from (**a**). Each row in (**b**) is called a record. For each record, the key of the record is the ID of the GO term, and the value of the record is a link list that contains all of the S-values of the key. For each term in (**a**), there is a corresponding record in (**b**). We can obtain the essential information from the hash table directly instead of from the DAG in (**a**). The proposed strategy converts the storage form of information from the GO graph into hash tables to speed the calculation process

In this subsection, we take Wang’s method as an example to show how to speed the functional similarity calculation. The main idea is illustrated in Fig. 2. The proposed strategy comprises two main steps.

**Step one**: Establish a hash table for the Wang method

According to Eq. (9), measuring the semantic similarity between two terms A and *B* is only based on three parts: SV(A), SV(B) and the numerator of Eq. (9). With further analysis, we fortunately find that the values of SV(A) and SV(B) are only dependent on the S-values for all terms in DAG_A_ and the S-values for all terms in DAG_B_, respectively. Besides, the numerator of Eq. (9) can also be calculated quickly based on the S-values of terms A and B.

From the analysis above, *S*_*A*_(*t*), *t* ∈ *T*(*A*) and *S*_*B*_(*t*), *t* ∈ *T*(*B*) are essential information for Eq. (9). To adopt the proposed strategy for calculating Eq. (9), we can compute all of the S-values of A and B in their corresponding DAGs and then store the results into a hash table. The S-values for GO:0043231 are listed in Table 3. The hash table is established based on Fig. 2a.

**Table 3:** S-values for GO terms in the DAG for *intracellular membrane-bound organelle*: 0043231

GO terms	0043231	0043229	0043227	0005622
S-value	1.0	0.8	0.8	0.48
GO terms	0005623	0043226	0005575	
S-value	0.288	0.64	0.512	

#### **Step two**: Measure the gene functional similarity based on the hash table

Once the hash table is established, measuring the semantic similarity is based on the hash table instead of the GO graph. In other words, because the hash table contains all of the essential information that Eq. (9) needs, the Wang method can obtain the information needed directly from the hash table. After obtaining the semantic similarity between GO pairs, it can use the BMA rule to further measure the functional similarity between two genes.

In Fig. 2a represents a DAG for the GO term *Intracellular Membrane*-*bound Organelle*: 0043231, and (b) represents the hash table established on the basis of (a). The semantic contribution factors for the ‘is-a’ and ‘part-of’ relations are 0.8 and 0.6, respectively. We use Eq. (7) to calculate all of the S-values for term GO:0043231 and list the results in Table 3. The storage form for term GO:0043231 is listed in the last row of Fig. 2b. For the other terms in Fig. 2a, the handling process is similar to term GO:0043231. The results are also listed in Fig. 2b. It is noteworthy that the order of records shown in Fig. 2b may differ from the actual order because of the special storage features of hash tables.

### Example: Measuring the semantic similarity for the Wang method

We take two terms GO:0043227, named *A*, and GO:0005622, named *B*, as an example to measure the semantic similarity based on the hash table. The relationship between *A* and *B* can be obtained from Fig. 2a. The semantic similarity of the two terms *A* and *B* is calculated in three steps based on the hash table as follows:

$$ SV(A)=1.0+0.8+0.64=2.44 $$

$$ SV(B)=1.0+0.6+0.48=2.08 $$

$$ {\displaystyle \sum_{t\in {T}_A\cap {T}_B}\left({S}_A(t)+{S}_B(t)\right)}={S}_A(0005575)+{S}_B(0005575)=0.48+0.64=1.12 $$

Therefore, the semantic similarity between terms *A* and *B* is:

$$ {S}_{GO}\left(A,B\right)=\frac{{\displaystyle \sum_{t\in {T}_A\cap {T}_B}\left({S}_A(t)+{S}_B(t)\right)}}{SV(A)+SV(B)}=\frac{1.12}{2.44+2.08}=0.25 $$

Because the S-values of terms *A* and *B* can be obtained directly from the hash table represented in Fig. 2b, there is no need to search the DAGs of terms *A* and *B*. Therefore, the computational efficiency of measuring the semantic similarity has sharply improved. The proposed strategy has achieved the desired result.

**Fig. 3:**
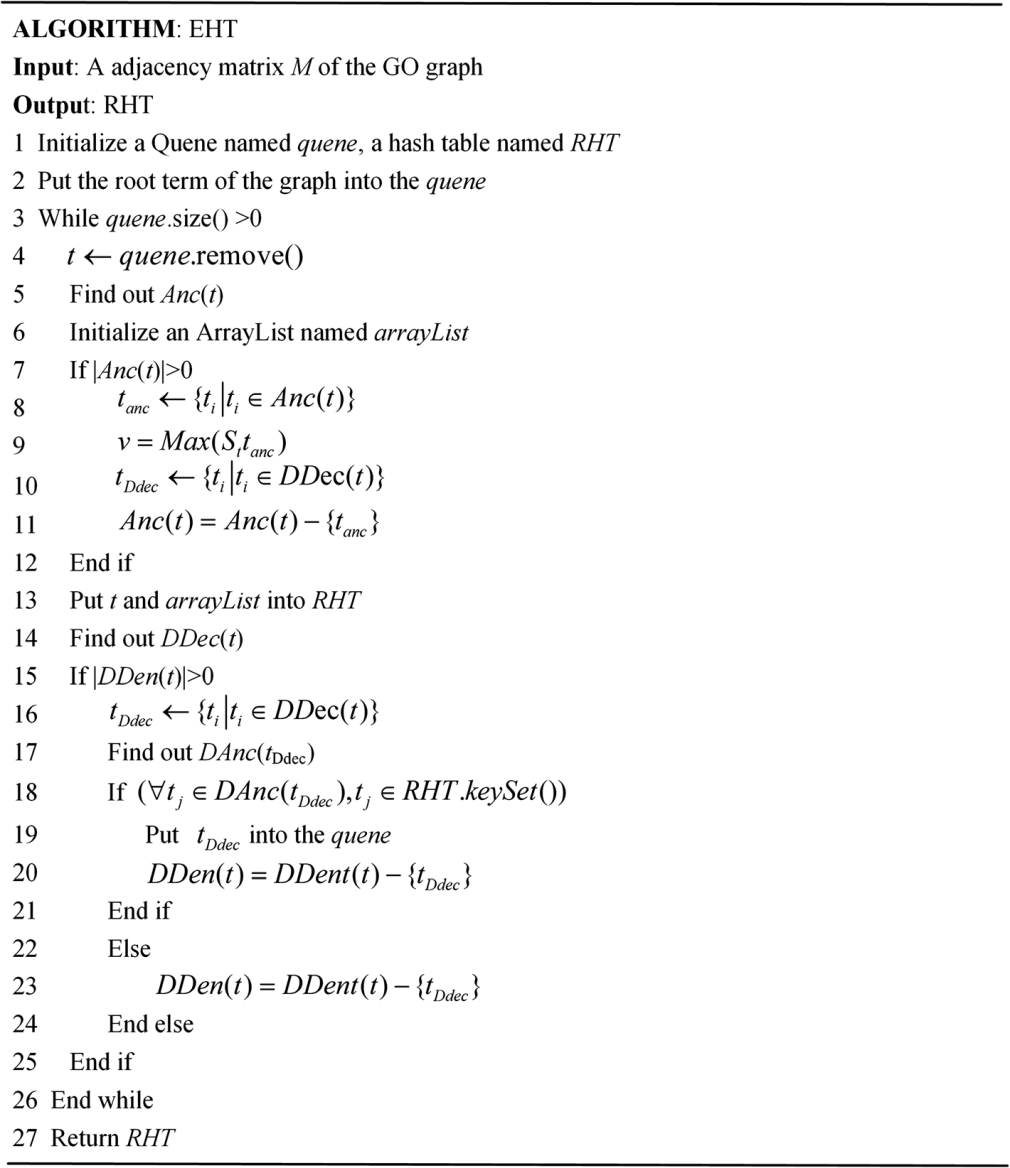
The algorithm for establishing a hash table from the GO graph for the Wang method

To obtain the corresponding hash table from the GO graph for the Wang method, we design an algorithm for establishing the hash table, namely EHT. The algorithm is described in Fig. 3. To simplify the description of the algorithm, we briefly explain the notations used in the algorithm. An adjacency matrix *M* represents one of three GO graphs MF, BP and CC. RHT represents the hash table that stores the essential information extracted from the GO graph. DDec(*t*) represents a set that contains all of the direct descendants of term *t*, and DAnc(*t*) represents a set that contains all of the direct ancestors of term *t*.

### Complexity analysis

Without loss of generality, suppose there are *m* pairs of genes that require computation of their functional similarities. Each gene has been annotated by an average *k* GO terms. There are *n* GO terms in the GO graph totally. Each pairwise approach uses the *BMA* rule to measure gene functional similarity. The time complexity for the seven methods is listed in Table 4.

**Table 4:** Time complexity for measuring gene functional similarity of each method

Method	Time complexity with the proposed strategy		Time complexity without the proposed strategy
	Step one	Step two	
Resnik	O(n^3^)	O(m*k^2^*n*logn)	O(m*n^3^*k^2^*n*logn)
Jiang	O(n^3^)	O(m*k^2^*n*logn)	O(m*n^3^*k^2^*n*logn)
Lin	O(n^3^)	O(m*k^2^*n*logn)	O(m*n^3^*k^2^*n*logn)
Pekar	O(n^4^)	O(m*k^2^*n*logn)	O(m*n^4^*k^2^*n*logn)
Wang	O(n^4^)	O(m*k^2^*n*logn)	O(m*n^4^*k^2^*n*logn)
simUI	O(n^3^)	O(m*k^2^*n*logn)	O(m*n^3^*k^2^*n*logn)
simGIC	O(n^3^)	O(m*k^2^*n*logn)	O(m*n^3^*k^2^*n*logn)

In step one, we can find that the time complexity for each method has no relationship with *m*. The time complexity for establishing the hash table is relatively low. In step two, once the hash tables are established, the efficiency of measurement of the gene functional similarity will be improved. It is noteworthy that the total time complexity for each gene functional similarity method equals the time complexity of step two as long as the GO graph remains unchanged. To further differentiate SGFSC from other tools in terms of time complexity, we also list the time complexity when our proposed strategy is not adopted. From the Table 4, we can find that, if *m* ≫ *n*, SGFSC has a higher computational efficiency comparing with other methods that don’t adopt the proposed strategy.

Regarding space complexity, the proposed strategy occupies some memory space to store the hash tables. Suppose the storage space of a GO term is *l*, then the space complexity for storing the hash table is O(n*k*l). The actual amount of memory required to store the hash table is about 5 MB, which has been verified through experimentation. There is no doubt that it will be quicker to store and read the hash table on a laptop computer.

## Results

In this section, we provide the running time to establish the hash table from the GO graph first. Then by using the proposed strategy, the time needed to measure the semantic similarity between term pairs is listed in Table 5. The results of measuring gene functional similarities on the whole genomic scale for model organisms are listed in Table 6. The results of comparisons with other tools are listed in Tables 7 and 8.

**Table 5:** Time in seconds required to establish the hash table for each method on BP, CC and MF ontologies

Type	Resnik	Pekar	Wang	simUI	simGIC
BP	441	876	181	84	562
CC	264	46	2.3	1.6	267
MF	379	179	4.9	4.7	384

**Table 6:** Running time to measure semantic similarity between term pairs for three ontologies

Method	# of term pairs	BP		MF		CC	
		# of related terms	Time (s)	# of related terms	Time (s)	# of related terms	Time (s)
Resnik	10^6^	27,864	5.9	9,943	2.5	3,817	4.4
Jiang	10^6^	27,864	6.7	9,943	2.9	3,817	3.8
Lin	10^6^	27,864	6.0	9,943	2.7	3,817	3.6
Wang	10^6^	27,864	6.2	9,943	2.6	3,817	4.1
Pekar	10^6^	27,864	5.8	9,943	2.6	3,817	3.7

**Table 7:** Time in seconds to measure gene functional similarity of five organisms

Organism	Type	# of annotated genes	# of average annotations	# of gene pairs	Time Resnik	Time Jiang	Time Lin	Time Wang	Time Pekar	Time simUI	Time simGIC
Human	BP	39337	4.35	7.74 × 10^8^	36702	36487	36366	36427	36972	19088	19762
	CC	35975	2.56	6.47 × 10^8^	5843	5815	5814	5810	5869	4921	5815
	MF	38404	2.32	7.37 × 10^8^	323	326	327	321	326	4519	4828
Arabidopsis	BP	25532	3.24	3.26 × 10^8^	6945	6957	5948	6956	6953	5238	5590
	CC	17683	2.00	1.56 × 10^8^	812	808	841	816	816	962	989
	MF	20305	1.92	2.06 × 10^8^	632	616	619	622	628	1048	1091
Yeast	BP	5906	3.20	1.74 × 10^7^	578	580	586	583	579	353	383
	CC	5660	2.22	1.60 × 10^7^	143	146	145	146	144	432	142
	MF	5902	2.30	1.74 × 10^7^	66	64	66	65	64	94	100
Rat	BP	23319	4.62	2.65 × 10^8^	13650	13690	13641	13714	13705	7219	7954
	CC	22217	2.60	2.47 × 10^8^	2319	2314	2299	2411	2319	1911	2083
	MF	23065	2.49	2.66 × 10^8^	1334	1315	1351	1339	1330	1750	1874
Oryza	BP	1909	1.44	1.82 × 10^6^	8	8	7	8	8	13	13
	CC	39995	1.06	8.00 × 10^8^	1262	1343	1307	1258	1254	2976	3115
	MF	2041	1.60	2.08 × 10^6^	5	5	5	4	5	9	10

**Table 8:** Running time in seconds for each tool to measure semantic similarity

Tool	# of term pairs		
	10^2^	10^4^	10^6^
SGFSC	<1	2	9.4
GFSAT	68	6,387	X
GOSemSim	52	3,634	X

### Running time to establish hash tables for each method

The key goal of the strategy is to extract the essential information from the GO graph and then establish a hash table to replace the GO graph. Therefore, we provide the running times to establish the hash table for each method in Table 9.

**Table 9:** Running time in seconds for each tool to measure gene functional similarity

Tool	# of gene pairs		
	10^2^	10^4^	10^6^
SGFSC	<1	29	768
GFSAT	163	36,514	X
GOSemSim	78	13,056	X

From the results, we can find that SGFSC can establish the hash table within a few minutes. For example, the running times for the Resnik method on BP, CC and MF ontologies are 441 s, 264 s and 379 s, respectively. The running times of the other methods are close to those of the Resnik method, indicating that the computational efficiency of the proposed strategy for establishing the hash table is high. SGFSC is efficient in updating the content of the hash tables. Therefore, SGFSC is well adapted to the daily evolution of the GO database, which may change with the deletion of obsolete terms and the addition of new terms. It should be noted that the hash tables for the Resnik, Lin and Jiang methods are the same. The experiments were conducted on Windows with an i5-2600 K CPU @ 3.30 GHz with 16 GB memory.

### Running time to measure semantic similarity for each method

**Fig. 4:**
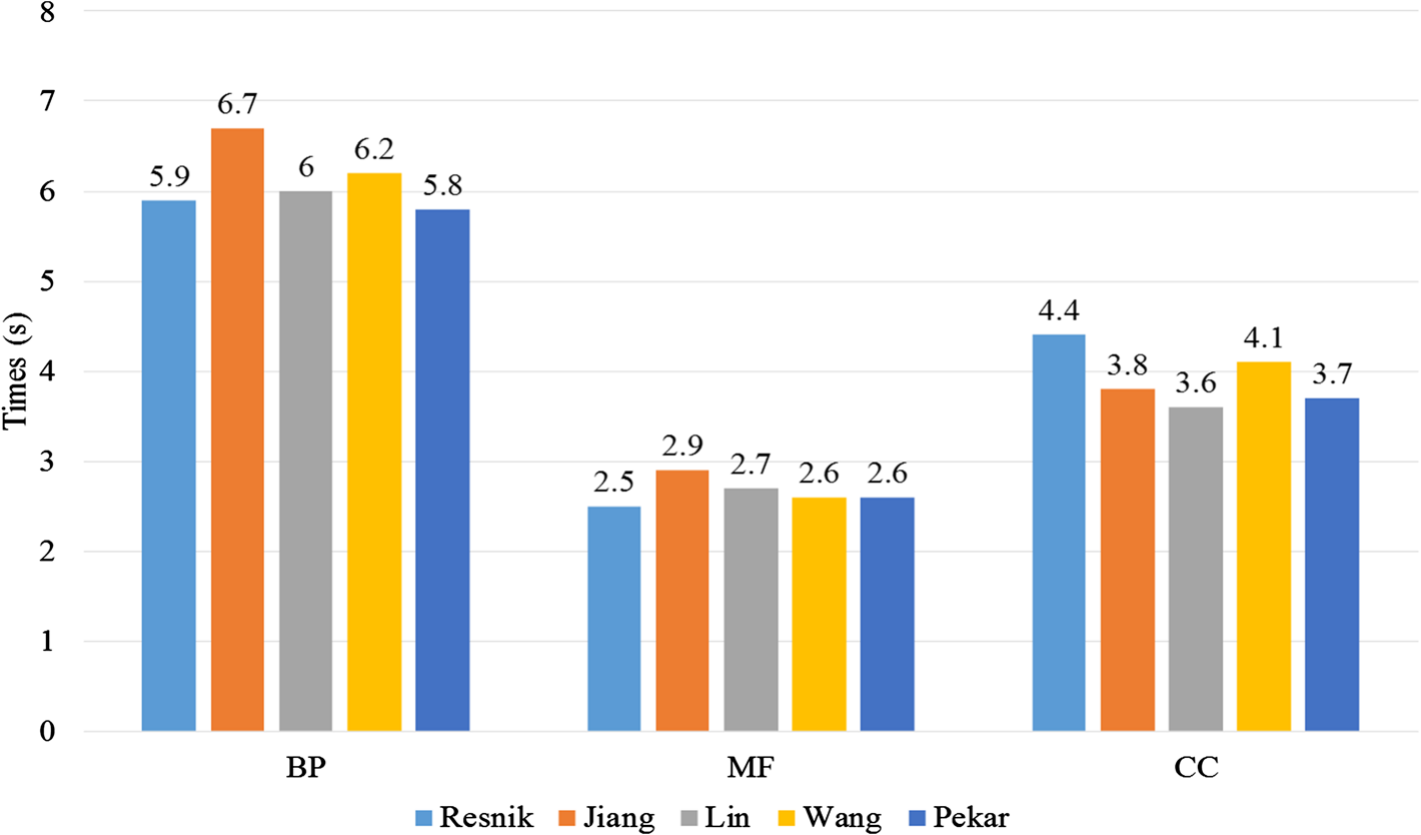
Bar plots of running time in measuring semantic similarity between term pairs

For pairwise approaches, measuring the functional similarity between genes is mainly dependent on the sematic similarity between term pairs. Therefore, the computational efficiency of sematic similarity plays a key role in the pairwise methods. We randomly select 10^6^ term pairs that are related to all of the terms in the corresponding GO graphs for the BP, MF and CC ontologies. The computing time for each method on BP, MF and CC graphs is listed in Table 5. For example, the running times for the Wang method on BP, CC and MF ontologies are 5.9 s, 2.5 s and 4.4 s, respectively. Because the BP ontology has the most terms, its running time is longer than that of the MF and CC ontologies. The results show that SGFSC can complete the calculation within a few seconds. In addition, the bar plots of running time for each method are presented in Fig. 4. We can clearly find that the computation time is within 10s.

### Running time to measure gene functional similarity for each method

To give a comprehensive comparison of the computational efficiency of each method, we select annotation data of five organisms representing Human, Arabidopsis, Yeast, Rat and Oryza. In Table 6, the number of annotated genes, number of average annotations for each gene, number of gene pairs and the running time in seconds for seven typical methods are presented for the corresponding ontologies of BP, CC and MF of five organisms.

The experiments select annotation data for five model organisms: Human, Arabidopsis, Yeast, Rat and Oryza. The UniProt-GOA data for each species were downloaded from http://geneontology.org/page/download-annotations in August 2015. Number of gene pairs refers to the number of gene pairs that will be measured for gene functional similarity using SGFSC. We obtain the gene pairs by combining all of the annotated genes in the GOA database. The experiments were conducted on Linux with an E5-2609 CPU @2.40 GHz and 64 GB memory.

For each organism, SGFSC computes the respective functional similarity on BP, CC and MF ontologies. For the whole genomic scale of human, the experimental results show that the computing time for each method is no more than 11 h. For the other organisms, the computing time is shorter (within a few hours) because the number of gene pairs and the number of average annotations of genes are relatively smaller. For example, the number of annotated genes for the human in BP ontology is 39337, whereas that for yeast is 5906. Therefore, the running time for the two organisms is greatly different. However, the running times of SGFSC for all selected organisms are within an acceptable range. Therefore, SGFSC shows its outstanding advantage for measuring functional similarity on the genomic scale. Even more, it has the ability to measure the similarity of a combination of all annotated genes in the GOA database for the model organisms. The proposed strategy thus can effectively solve the problem of large-scale computing of gene functional similarity.

**Fig. 5:**
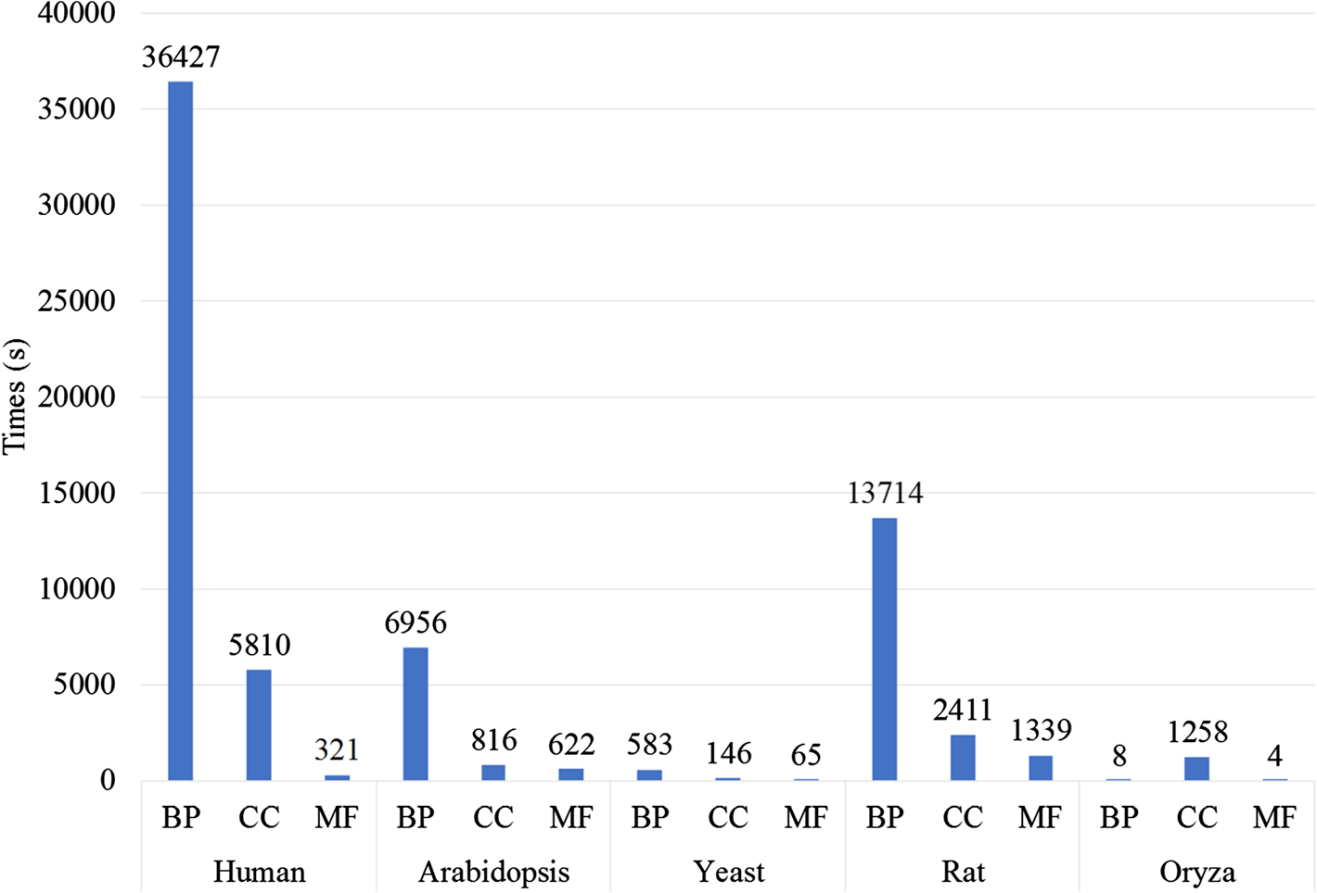
Bar plots for running time in measuring gene functional similarity using Wang method on each organism

**Fig. 6:**
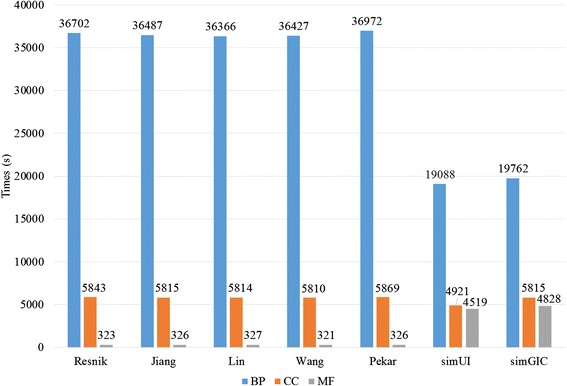
Bar plots for running time for each method in measuring functional similarity on human genomic scale

To represent the experimental results more intuitive, we add two bar plots which are depicted using Figs. 5 and 6. Figure 5 is the bar plots for running time using Wang method on selected organisms. Wang method can finish the gene functional similarity calculation on the genome scale for all selected organism in a relatively short time. Figure 6 shows the running time for each selected method on human genome scale. Results also indicates that all these methods can complete the calculation in an acceptable period of time.

### Comparison with other tools

To compare the computational efficiency of SGFSC with other tools, we select two tools: GFSAT [23] and GOSemSim [33]. These two tools can be conveniently installed on a laptop, and therefore, we can accurately measure running times. It is difficult to accurately measure the running times of other tools because they can only be used online. Therefore, we decided to compare running time of these two tools only.

We use the running time to compute the similarities of a large number of term pairs and gene pairs to evaluate the computational efficiency of these three tools. In the experiment, we use SGFSC, GFSAT and GOSemSim to compute the similarities of three sets of term pairs and gene pairs, respectively. The numbers of term pairs and gene pairs in these sets are both 10^2^, 10^4^ and 10^6^, respectively. The experiments were conducted on Windows with an i5-2600 K CPU @ 3.30 GHz and 16 GB memory.

The GO data in Table 7 were downloaded in August 2015, and the term pairs were randomly generated from the MF ontology. The Wang method was used for each tool. The GOA data in Table 8 of Arabidopsis was downloaded in August 2015. We selected the well-annotated genes and then generated gene pairs for testing. The Wang method was also used for each tool. ‘X’ in Tables 7 and 8 indicates that the process took >12 h.

The running time of each tool to measure the semantic similarity results is listed in Table 7. The running times of GFSAT and GOSemSim to measure the semantic similarity of 10^4^ term pairs were 6387 s and 3634 s, respectively, whereas that for SGFSC took only 2 s. Furthermore, the running times of both GFSAT and GOSemSim to calculate 10^6^ term pairs were greater than 12 h, whereas that for SGFSC was only 9.4 s.

**Fig. 7:**
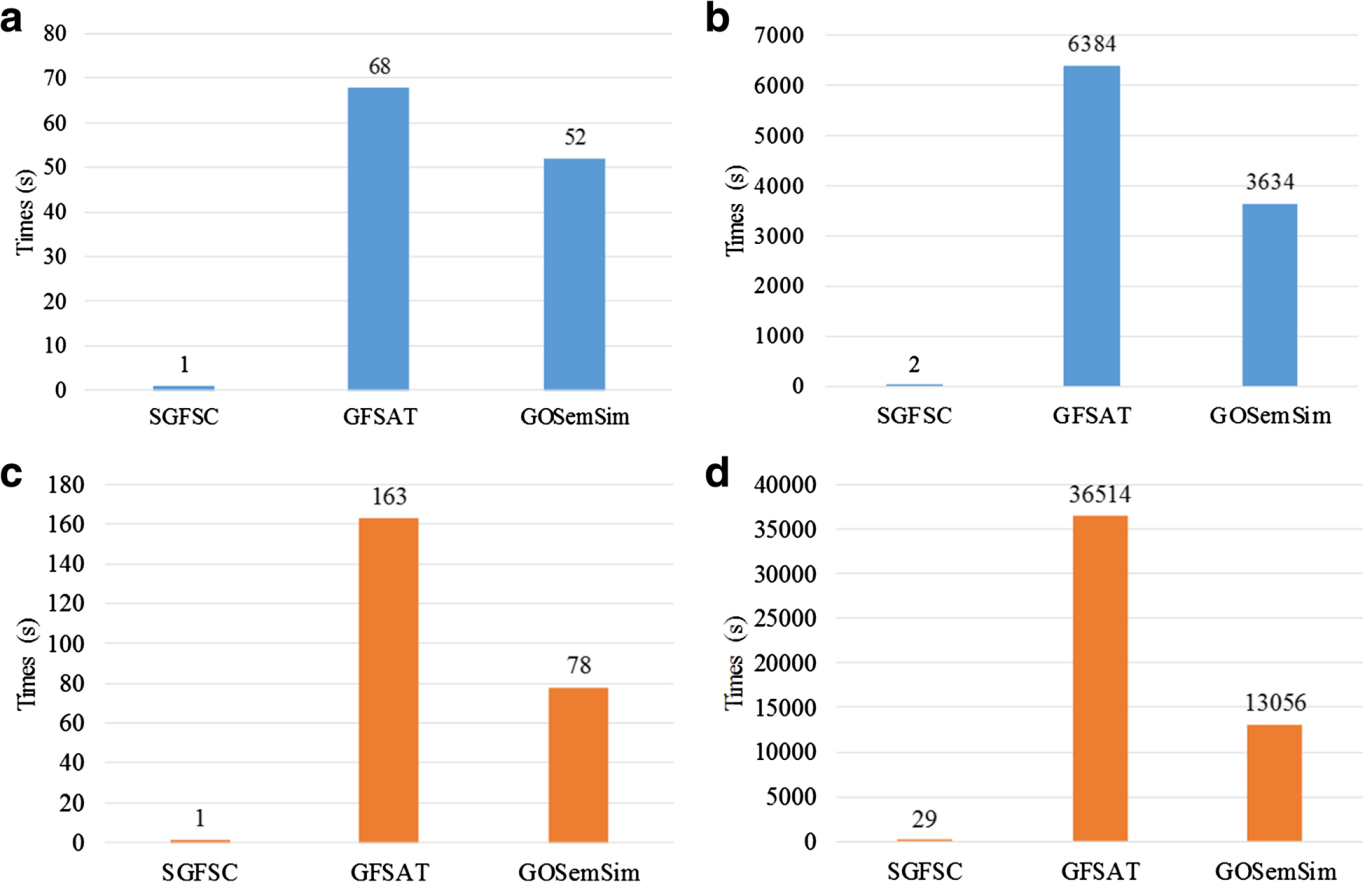
Bar plots for running time on different datasets using SGFSC, GFSAT and GOSemSim

The running time of each tool to measure gene functional similarity is listed in Table 8. Similar to the findings for semantic similarity, SGFSC performed considerably better than the other two tools on the similarity calculation of gene pairs. The running time of SGFSC was 29 s for 10^4^ gene pairs, whereas GFSAT and GOSemSim required 36154 s and 13056 s, respectively. As the results clearly show, in comparison with the other two tools, SGFSC has a considerable speed advantage especially when calculating a large number of gene pairs. Besides, we add one merged bar plots Figure for Tables 7 and 8. Figure 7a and b show the running time of measuring semantic similarity on 10^2^ and 10^4^ term pair datasets. Figure 7c and d depict the running time of measuring gene functional similarity on 10^2^ and 10^4^ gene pair datasets respectively. All of the four subfigures clearly show the advantages of SGFSC in the computation time. Therefore, our proposed strategy achieves the desired results in speeding up the gene functional similarity calculation.

## Discussion

First, we emphasise that the aim of the proposed two-step strategy is only to speed up the methods used to measure gene functional similarity because these methods tend to be time consuming if they are not implemented with a proper data structure. The problem may be extremely prominent especially when gene functional similarity needs to be measured on the genome scale for some applications. Therefore, the proposed strategy for speeding up the functional similarity calculation is quite meaningful.

Second, the proposed two-step strategy adopts hash tables as the data structure to store essential information to avoid traversing the GO graph. Furthermore, the hash table is used only to satisfy the requirement of a quick search. Hence, the computational efficiency of these methods improves significantly. We particularly highlight that the computational efficiency for these methods varies widely according to different data structures and implementation strategies. Therefore, it is critical to choose an appropriate implementation strategy to increase the computational efficiency of these methods.

Third, we also implemented an online tool, SGFSC, for adoption of the two-step strategy. The SGFSC could speed the functional similarity calculation methods on the whole genomic scale. However, there are some other issues need to note.

### Precision

SGFSC extracts the essential information for each method from the GO graph and then establish hash tables, which have a special structure to store the information. Therefore, the precision of the final computing result is not lost with SGFSC because the function of the hash tables is only to store intermediate results to avoid repeatedly traversing the GO graph for each method.

### Correctness

SGFSC was implemented with seven typical methods. It is not difficult to understand that SGFSC only achieves the computation speeds with these methods when our proposed two-step strategy is used. The computing process of the original methods is unchanged. Therefore, the computing results of SGFSC are identical to those of other tools if the same versions of the GO and GOA databases are used. As a result, the computing results are absolutely correct.

### Space

Because SGFSC adopts a two-step computing strategy, it first needs to read the hash table into memory. Therefore, it occupies some additional memory space compared with other tools that do not adopt the proposed strategy. Experimentation showed that the memory space for storing the hash table is about 5 MB. The continuing development of computer hardware technology has made this a simple problem to solve. The core idea of the strategy comes from making the best use of memory space to reduce the computing time, which is widely used in computer science.

### Application

SGFSC offers powerful computational capability to compute gene functional similarity on a genomic scale. We have provided a friendly online tool for the convenient use of SGFSC. In addation, our proposed strategy also offers good versatility for use in other research. For example, in the area of natural language process research, because the size of the WordNet is large, computing the IC value of a concept is also time consuming. Therefore, researchers measuring the semantic similarity between concepts could adopt our proposed two-step strategy to improve computational efficiency.

### Drawback

To speed up the gene functional similarity calculation, SGFSC first has to read the corresponding information hash table into memory, which will take a few seconds. Therefore, the computational efficiency of SGFSC is lower than that of other tools if the number of gene pairs requiring measurement of functional similarity is small. Therefore, the outstanding advantage of SGFSC is in its measurement of gene functional similarity for a large number of gene pairs.

## Conclusion

First, a novel two-step computing strategy is proposed to speed up gene functional similarity calculation. These methods measure gene functional similarity based on hash tables. Therefore, the time complexity is obviously decreased because there is no need to traverse the GO graph repeatedly, which primarily affects the computational efficiency.

Second, we have implemented an online tool called SGFSC that is bundled with seven typical gene functional similarity calculation methods and is freely available at http://nclab.hit.edu.cn/SGFSC. The computational efficiency of SGFSC offers a significant improvement in computing time. Our experiments show that SGFSC has a great advantage in measuring gene functional similarity on the whole genomic scale.

Third, the key point in our proposed strategy is the transformative idea that the information required can be obtained directly from the hash tables instead of the original GO graph. The proposed strategy converts the storage form of information from a GO graph into a hash table structure that can meet the requirements of a quick query. As a result, the proposed strategy achieved a desired result. The proposed strategy may also be applied to other areas of bioinformatics to improve computational efficiency.

## References

[1] Brameier M, Wiuf C (2007). Co-clustering and visualization of gene expression data and gene ontology terms for Saccharomyces cerevisiae using self-organizing maps. J Biomed Inform.

[2] Cho YR, Zhang AD, Xu X (2009). Semantic similarity based feature extraction from microarray expression data. Int J Data Min Bioinform.

[3] Yang D, Li YH, Xiao H, Liu Q, Zhang M, Zhu J, Ma WC, Yao C, Wang J, Wang D (2008). Gaining confidence in biological interpretation of the microarray data: the functional consistence of the significant GO categories. Bioinformatics.

[4] Qu Y, Xu S (2004). Supervised cluster analysis for microarray data based on multivariate Gaussian mixture. Bioinformatics.

[5] Li D, Liu W, Liu Z, Wang J, Liu Q, Zhu Y, He F (2008). PRINCESS, a protein interaction confidence evaluation system with multiple data sources. Mol Cell Proteomics.

[6] Jain S, Bader GD (2010). An improved method for scoring protein-protein interactions using semantic similarity within the gene ontology. BMC Bioinformatics.

[7] Schlicker A, Huthmacher C, Ramírez F, Lengauer T, Albrecht M (2007). Functional evaluation of domain-domain interactions and human protein interaction networks. Bioinformatics.

[8] Guzzi PH, Mina M, Guerra C, Cannataro M (2012). Semantic similarity analysis of protein data: assessment with biological features and issues. Brief Bioinform.

[9] Ortutay C, Vihinen M (2009). Identification of candidate disease genes by integrating Gene Ontologies and protein-interaction networks: case study of primary immunodeficiencies. Nucleic Acids Res.

[10] Nariai N, Kolaczyk ED, Kasif S (2007). Probabilistic protein function prediction from heterogeneous genome-wide data. PLoS One.

[11] Yu G, Zhu H, Domeniconi C, Liu J (2015). Predicting protein function via downward random walks on a gene ontology. BMC Bioinformatics.

[12] Guangyuan Fu, Wang J, Yang B, Yu G, Guangyuan Fu, Wang J, Yang B, Guoxian Yu (2016). NegGOA: negative GO annotations selection using ontology structure. Bioinformatics.

[13] Cheng L, Li J, Ju P, Peng J, Wang Y (2014). SemFunSim: a new method for measuring disease similarity by integrating semantic and gene functional association. PLoS One.

[14] Chen J, Aronow BJ, Jegga AG (2009). Disease candidate gene identification and prioritization using protein interaction networks. BMC Bioinformatics.

[15] Schlicker A, Lengauer T, Albrecht M (2010). Improving disease gene prioritization using the semantic similarity of Gene Ontology terms. Bioinformatics.

[16] Teng Z, Guo M, Liu X, Dai Q, Wang C, Xuan P (2013). Measuring gene functional similarity based on group-wise comparison of GO terms. Bioinformatics.

[17] Schlicker A, Domingues FS, Rahnenführer J, Lengauer T (2006). A new measure for functional similarity of gene products based on Gene Ontology. BMC Bioinformatics.

[18] Jiang JJ, Conrath DW. Semantic similarity based on corpus statistics and lexical taxonomy. arXiv preprint cmp-lg/9709008 1997.

[19] Lin D. An information-theoretic definition of similarity. In: ICML. 1998, Vol.98:296–304. https://scholar.google.com/scholar?q=An+information-theoretic+definition+of+similarity&btnG=&hl=zh-CN&as_sdt=0%2C5.

[20] Resnik P (1999). Semantic similarity in a taxonomy: An information-based measure and its application to problems of ambiguity in natural language. J Artif Intell Res.

[21] Wang JZ, Du Z, Payattakool R, Philip SY, Chen CF (2007). A new method to measure the semantic similarity of GO terms. Bioinformatics.

[22] Pesquita C, Faria D, Bastos H, Ferreira AE, Falcão AO, Couto FM (2008). Metrics for GO based protein semantic similarity: a systematic evaluation. BMC Bioinformatics.

[23] Xu Y, Guo M, Shi W, Liu X, Wang C (2013). A novel insight into Gene Ontology semantic similarity. Genomics.

[24] Bandyopadhyay S, Mallick K (2014). A new path based hybrid measure for Gene Ontology similarity. IEEE/ACM Trans Comput Biol Bioinform.

[25] Song X, Li L, Srimani PK, Yu PS, Wang JZ (2014). Measure the semantic similarity of GO terms using aggregate information content. IEEE/ACM Trans Comput Biol Bioinform.

[26] Wu H, Su Z, Mao F, Olman V, Xu Y (2005). Prediction of functional modules based on comparative genome analysis and Gene Ontology application. Nucleic Acids Res.

[27] Cheng J, Cline M, Martin J, Finkelstein D, Awad T, Kulp D, Siani-Rose MA (2004). A knowledge-based clustering algorithm driven by gene ontology. J Biopharm Stat.

[28] Li M, Wu X, Pan Y, Wang J (2013). hF-measure: A new measurement for evaluating clusters in protein-protein interaction networks. Proteomics.

[29] Smyth GK: Limma: linear models for microarray data. Bioinformatics and computational biology solutions using R and Bioconductor Springer. 2005;397–420. https://scholar.google.com/scholar?q=Bioinformatics+and+computational+biology+solutions+using+R+and+Bioconductor+Springer&btnG=&hl=zh-CN&as_sdt=0%2C5.

[30] Pekar V, Staab S: Taxonomy learning: factoring the structure of a taxonomy into a semantic classification decision. In: Proceedings of the 19th International Conference on Computational linguistics-Volume 1: 2002. Association for Computational Linguistics: 1–7.

[31] Pesquita C, Faria D, Falcão AO, Lord P, Couto FM (2009). Semantic similarity in biomedical ontologies. PLoS Comput Biol.

[32] Harispe S, Ranwez S, Janaqi S, Montmain J (2014). The semantic measures library and toolkit: fast computation of semantic similarity and relatedness using biomedical ontologies. Bioinformatics.

[33] Yu G, Li F, Qin Y, Bo X, Wu Y, Wang S (2010). GOSemSim: an R package for measuring semantic similarity among GO terms and gene products. Bioinformatics.

[34] Faria D, Pesquita C, Couto F, Falcao A: ProteInOn: A Web Tool for Protein Semantic Similarity. DI/FCUL TR 07-6, Department of Informatics, University of Lisbon. 2007. [http://www.di.fc.ul.pt/techreports/07-6.pdf]. https://scholar.google.com/scholar?q=+ProteInOn%3A+A+Web+Tool+for+Protein+Semantic+Similarity&btnG=&hl=zh-CN&as_sdt=0%2C5.

[35] Mazandu GK, Mulder NJ (2013). Information content-based gene ontology semantic similarity approaches: toward a unified framework theory. Biomed Res Int.

[36] Du Z, Li L, Chen CF, Philip SY, Wang JZ (2009). G-SESAME: web tools for GO-term-based gene similarity analysis and knowledge discovery. Nucleic Acids Res.

[37] Jiang R, Gan MX, He P (2011). Constructing a gene semantic similarity network for the inference of disease genes. BMC Syst Biol.

[38] Mordelet F, Vert JP (2011). ProDiGe: Prioritization of Disease Genes with multitask machine learning from positive and unlabeled examples. BMC Bioinformatics.

[39] Yang P, Li XL, Mei JP, Kwoh CK, Ng SK (2012). Positive-unlabeled learning for disease gene identification. Bioinformatics.

[40] Wu S-Y, Shao F-J, Sun R-C, Sui Y, Wang Y, Wang J-l (2014). Analysis of human genes with protein-protein interaction network for detecting disease genes. Physica A: Statistical Mechanics and its Applications.

[41] Yang P, Li X, Chua HN, Kwoh CK, Ng SK (2014). Ensemble positive unlabeled learning for disease gene identification. PLoS One.

[42] Pesquita C, Faria D, Bastos H, Falcão A, Couto F. Evaluating GO-based semantic similarity measures. In: Proceedings of the 10th Annual Bio-Ontologies Meeting: 2007. 38.

